# Evaluating Troponin-Based Monitoring in Patients Undergoing Immune Checkpoint Inhibitor Therapy

**DOI:** 10.1016/j.jacadv.2024.101374

**Published:** 2024-11-07

**Authors:** Yudai Tamura, Yuichi Tamura

**Affiliations:** a Pulmonary Hypertension Center, International University of Health and Welfare Mita Hospital, Tokyo, Japan; b Cardiology, Kahoku Central Hospital, Ishikawa, Japan; c Department of Cardiology, International University of Health and Welfare School of Medicine, Narita, Japan

**Keywords:** immune checkpoint inhibitor, immune-related adverse events, myocarditis, troponin

**Figure:**
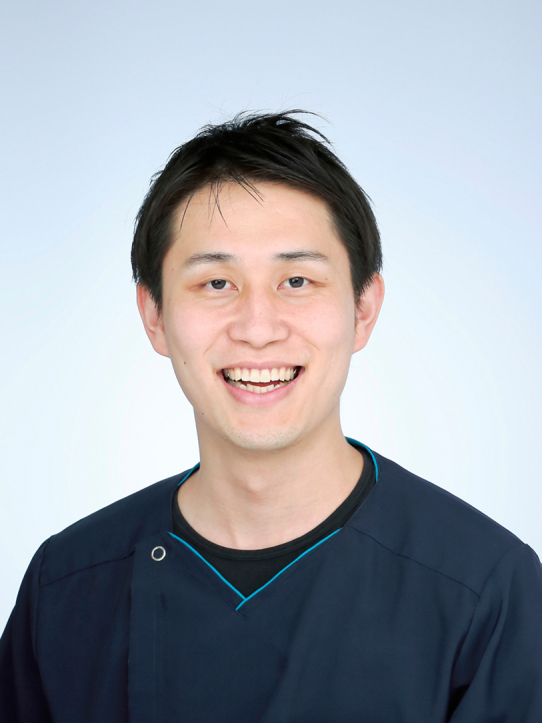

Immune checkpoint inhibitors (ICIs) have been effective in reducing mortality and recurrence risk for various cancers, leading to their expanded use. However, with the increased use of ICIs, there is a growing concern about immune-related adverse events (irAEs). Among the most severe irAEs is ICI-associated myocarditis. Although the incidence of ICI-associated myocarditis has been reported to be low at approximately 1%, its mortality rate is notably high, ranging from 30% to 50%.^1^, ^2^, ^3^ Early treatment interventions for ICI-associated myocarditis have been shown to improve prognosis, emphasizing the importance of early detection and intervention.^4^

In 2022, the European Society of Cardiology published guidelines for cardio-oncology, recommending regular troponin measurements for patients receiving ICIs, with a Class Ⅱa recommendation.^5^ Patients considered high risk include those receiving dual ICIs (such as nivolumab and ipilimumab), ICIs combined with cardiotoxic drugs, ICIs with noncardiovascular irAEs (other organ irAEs), a history of cancer therapy-related cardiac dysfunction, or existing cardiovascular disease. Regardless of risk, it is recommended to measure troponin levels before the second, third, and fourth courses of ICI therapy, although it is noted that monitoring ICI therapy remains challenging due to the lack of evidence.

Certainly, troponin elevation is a fundamental criterion in diagnosing ICI-associated myocarditis. A registry reported that 94% of patients with ICI-associated myocarditis had elevated troponin levels.^1^ The primary advantage of troponin measurement is its simplicity, providing an opportunity to perform further diagnostic evaluations such as echocardiography or cardiac magnetic resonance imaging to differentiate ICI-associated myocarditis.

In a prospective single-center observational study conducted by Bracun et al,^6^ significant elevations in high-sensitivity troponin T (hs-TnT) were observed in 26 out of 164 patients (16%) treated with ICIs. Among these 26 patients, only 8 (5%) were diagnosed with myocarditis. Patients diagnosed with myocarditis had significantly higher hs-TnT levels compared to those who were not diagnosed. hs-TnT levels exceeded 160 ng/L in patients diagnosed with ICI-associated myocarditis but did not exceed 156 ng/L in those without myocarditis. The mortality rate among patients with ICI-associated myocarditis was 12.5%.

Similarly, a prospective observational study conducted in Japan found that among 129 patients treated with ICIs, 14% exhibited elevated high-sensitivity troponin I (hs-TnI) levels, and 4.7% were diagnosed with ICI-associated myocarditis, with a myocarditis mortality rate of 16.7%.^7^ These frequencies are consistent with the results of the aforementioned study. Prospective surveillance using troponin leads to a higher incidence of myocarditis compared to previous reports. However, a consistent finding is the lower mortality rate from myocarditis. This is likely due to the frequent screening that enables the diagnosis of ICI-associated myocarditis in its milder stages. Additionally, appropriate interventions before the condition becomes severe likely contribute to the decreased mortality rate. These studies indirectly indicate that early detection and appropriate management contribute to better outcomes.

The notable novelty of this study is the identification of a clinically significant troponin elevation cutoff. A single elevation in troponin levels may indicate mild myocarditis, which might not be clinically significant and may not be suggested by imaging diagnostics, with some cases only being diagnosed postmortem.^8^^,^^9^ Another concern is the potential overdiagnosis of clinically insignificant myocarditis triggered by troponin measurement. In other words, there may be cases where it is inappropriate to discontinue ICIs based solely on troponin elevation. Discontinuing ICIs could lead to cancer progression; hence, unnecessary discontinuation due to overdiagnosis of irAEs should be avoided. The Waliany et al^10^ report showed that out of 24 patients with elevated hs-TnI, only 3 had ICI-associated myocarditis. Similarly, our report found no patients in long-term ICI therapy discontinued treatment despite elevated hs-TnI.^11^ These findings suggest that detecting troponin elevation using general test upper limits and discontinuing ICIs is premature.

Additionally, studies investigating markers other than cardiac biomarkers, such as troponin, for diagnosing myocarditis and predicting prognosis have identified creatine kinase (CK) as the most effective marker.^12^ Our study also found that all patients who discontinued ICIs due to ICI-associated myocarditis had elevated CK levels.^13^ The relationship between CK elevation and clinically significant myocarditis has two backgrounds. First, CK elevation may originate from the myocardium, indicating severe myocarditis with extensive myocardial damage. Second, the main cause of CK elevation might be acute onset myositis or myasthenia gravis, frequently associated with myocarditis, referred to as “3M.” Particularly, myocarditis combined with myositis is associated with high mortality.^14^

To determine whether to discontinue ICI treatment and intervene for myocarditis, it is crucial to rapidly conduct a multimodal evaluation. Troponin elevation serves as the initial screening tool, but the extent of troponin elevation, exclusion of other conditions causing elevation (such as pulmonary embolism or type 2 myocardial infarction), and trends in other biomarkers like CK, along with readily available diagnostic tools like echocardiography and cardiac magnetic resonance imaging, must all be considered. Additionally, monitoring the trend of troponin levels is important. In the current study, the highest value of hs-TnT was used for analysis, rather than the initial detected value. This suggests that when a mild troponin elevation is first detected, frequent follow-up troponin measurements are necessary to determine whether the myocarditis is clinically significant. Detecting mild troponin elevation early allows time to monitor and assess the presence of myocarditis. In this regard, the study clarified the strengths and limitations of troponin-based surveillance in predicting and diagnosing ICI-associated myocarditis.

## Funding support and author disclosures

The authors have reported that they have no relationships relevant to the contents of this paper to disclose.
